# Nivolumab-Induced Hemophagocytic Lymphohistiocytosis (HLH) in a Patient With Metastatic Melanoma: A Case Report and Literature Review

**DOI:** 10.7759/cureus.95260

**Published:** 2025-10-23

**Authors:** Ravjot K Virdi, Umar Hussain, Jacob Bitran

**Affiliations:** 1 Internal Medicine, Advocate Lutheran General Hospital, Park Ridge, USA; 2 Hematology and Oncology, Advocate Lutheran General Hospital, Park Ridge, USA

**Keywords:** hemophagocytic lymphohistiocytosis, hyperinflammatory syndrome, immune checkpoint inhibitors, immune-related adverse events, immunotherapy

## Abstract

Immune checkpoint inhibitors (ICIs) have transformed cancer therapy, particularly in melanoma. However, their immune-enhancing mechanisms can lead to rare but severe immune-related adverse events (irAEs), including hemophagocytic lymphohistiocytosis (HLH), a hyperinflammatory syndrome marked by uncontrolled immune activation. HLH is challenging to diagnose due to symptom overlap with sepsis, disease progression, and other irAEs. This study presents a case of steroid-refractory HLH following nivolumab therapy and a literature review of published cases to highlight patterns in diagnosis, treatment, and outcomes. We conducted a chart review of a 79-year-old male patient with metastatic melanoma who developed HLH after nivolumab therapy at a community hospital in Park Ridge, Illinois. Additionally, a literature review (2018-2024) was performed using PubMed Central to identify HLH cases linked to ICIs. Inclusion criteria required clear reporting of the ICI used, underlying malignancy, time to HLH onset, diagnostic approach, treatment, and clinical outcomes. The patient developed HLH after his second nivolumab dose. Initial immunosuppressive therapy, including dexamethasone, tocilizumab, anakinra, and ruxolitinib, was ineffective. Improvement occurred only after initiating the HLH-2004 protocol with etoposide and adjunctive cyclosporine. His course was complicated by infections and neutropenia, but recovery was achieved after 10 weeks. The literature review included 16 publications. HLH typically develops within 1-6 weeks of ICI initiation, most often with nivolumab or pembrolizumab. While corticosteroids were first-line therapy, escalation to etoposide, intravenous immunoglobulin (IVIG), or cytokine inhibitors was common in severe cases. Approximately 70-75% of patients recovered. HLH is a rare but potentially fatal irAE of ICIs. Early recognition and aggressive treatment are essential. Oncology-specific diagnostic criteria and treatment guidelines are needed to improve outcomes as immunotherapy use expands.

## Introduction

Immune checkpoint inhibitors (ICIs) have transformed the management of numerous malignancies, including melanoma, non-small cell lung cancer, renal cell carcinoma, and hematologic neoplasms. These agents enhance antitumor immune responses by blocking inhibitory pathways such as PD-1, PD-L1, and CTLA-4, thereby promoting T-cell activation. Commonly used ICIs include nivolumab, pembrolizumab, and ipilimumab. Their integration into both adjuvant and metastatic treatment settings has led to substantial improvements in survival and disease control, revolutionizing modern oncology. 

Immune activation comes at the cost of immune-related adverse events (irAEs). These toxicities can affect virtually any organ system, with dermatologic, gastrointestinal, and endocrine manifestations being among the most common. Rare, but potentially life-threatening, irAEs include cytokine release syndrome (CRS), immune effector cell-associated neurotoxicity syndrome (ICANS), macrophage activation syndrome (MAS), and hemophagocytic lymphohistiocytosis (HLH). 

HLH is a hyperinflammatory syndrome driven by uncontrolled immune activation and excessive cytokine release. According to the HLH-2004 diagnostic criteria, HLH can be established if (A) there is a molecular diagnosis consistent with HLH, or (B) five of the eight following criteria are fulfilled: fever >38.5°C, splenomegaly, cytopenia affecting at least two lineages in peripheral blood, hypertriglyceridemia and/or hypofibrinogenemia, low or absent natural killer cell activity, serum ferritin concentration >= 500 ug/L, soluble interleukin-2 receptor >= 2400 units/mL, and presence of hemophagocytosis in bone marrow, spleen, liver, lymph nodes, or other tissues [1]. Diagnosis is particularly challenging, as HLH symptoms overlap with conditions such as sepsis, CRS, MAS, and malignancy-related inflammation. While the HLH-2004 criteria and H-score are commonly used diagnostic tools, both have limitations in adult cancer patients. The HLH-2004 criteria were originally developed for pediatric cases of genetic HLH and lack specificity in oncology populations. The H-score, although more adaptable, may yield false positives due to overlapping lab abnormalities in patients with cancer [2]. 

An increasing number of case reports and series have linked HLH to PD-1/PD-L1 inhibitors, with combination regimens (e.g., PD-1 plus CTLA-4 blockade) carrying heightened risk. Due to symptom overlap with other irAEs and malignancy progression, HLH remains underrecognized in the oncology setting. Early recognition and intervention are critical, as delays in diagnosis can result in rapid clinical deterioration, multiorgan failure, and death. 

In this report, we present a clinically significant case of nivolumab-induced HLH in a patient with metastatic melanoma. In addition to contributing to the limited but growing literature on this rare toxicity, we include a focused literature review to identify diagnostic challenges, treatment patterns, and outcomes associated with ICI-related HLH. 

## Case presentation

Case report

The authors encountered this clinical case of HLH at a community hospital in Park Ridge, Illinois, USA. Relevant clinical information was obtained through a comprehensive review of the patient’s electronic medical records. 

Patient Background 

A 79-year-old male patient with a history of stage IIb melanoma diagnosed in 2022 underwent surgical resection followed by initiation of adjuvant nivolumab in December 2024. He received his second dose in January 2025. One week later, he presented with shortness of breath, fatigue, and palpitations. Laboratory evaluation, summarized in Table 1, revealed a new isolated anemia with a hemoglobin level of 5.9 g/dL (baseline: 9-10 g/dL). Additional labs showed total bilirubin of 4.6 mg/dL, normal aspartate aminotransferase (AST)/alanine aminotransferase (ALT), lactate dehydrogenase (LDH) of 315 U/L, and haptoglobin <8 mg/dL. Direct Coombs and cold agglutinin tests were negative. Suspecting immune-mediated hemolysis from checkpoint inhibition, he was treated with dexamethasone and discharged on a steroid taper after hemoglobin improved to baseline. 

**Table 1:** Laboratory findings on initial presentation

Laboratory finding	Value	Reference range
Hemoglobin	5.9 g/dL	13-17 g/dL
Total bilirubin	4.6 mg/dL	0.2-1 mg/dL
Aspartate aminotransferase (AST)	25 units/L	<=37 units/L
Alanine aminotransferase (ALT)	37 units/L	<64 units/L
Lactate dehydrogenase (LDH)	315 units/L	86-234 units/L
Haptoglobin	<8 mg/dL	30-200 mg/dL
Direct Coombs	Negative	Negative
Cold agglutinin test	<1:32	<1:32

His medical history was also notable for stage 0 chronic lymphocytic leukemia (CLL) under surveillance, hypertension, hyperlipidemia, type 2 diabetes mellitus, chronic kidney disease stage 3, gastroesophageal reflux disease, benign prostatic hyperplasia, and obstructive sleep apnea managed with CPAP. 

Clinical Presentation and Diagnostic Evaluation 

**Figure 1:**
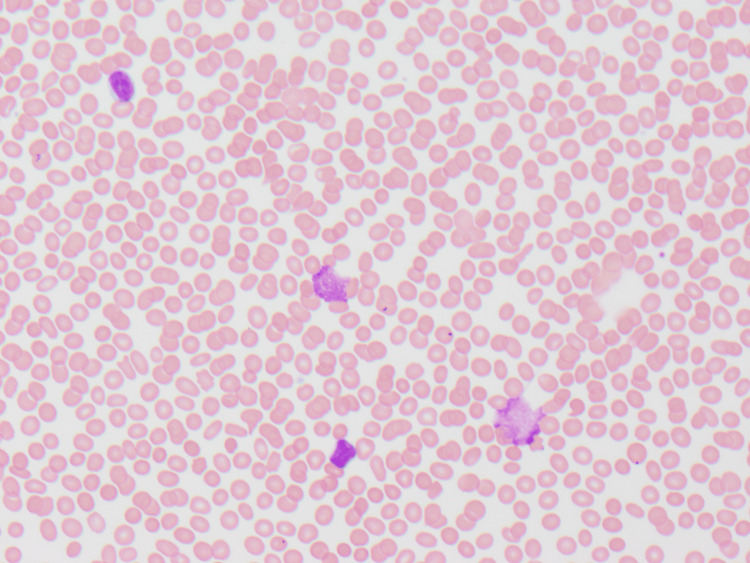
Peripheral smear with smudge cells and an absence of schistocytes

Three weeks after dose 2 of nivolumab, the patient re-presented with profound fatigue, occipital headaches, and neck pain. Labs (Table 2) revealed a new bicytopenia: hemoglobin 7.2 g/dL and platelets 31 K/mcL. Further evaluation showed an LDH of 623 U/L, haptoglobin <8 mg/dL, and a reticulocyte count of 0.3%. The peripheral smear (Figure 1) showed smudge cells but no schistocytes. Ferritin was markedly elevated at 5,344 ng/mL, D-dimer at 8.9 mg/L, and ALT was mildly elevated at 64 U/L. Direct and indirect Coombs tests, fecal occult blood, paroxysmal nocturnal hemoglobinuria panel, ADAMTS13 activity, and triglycerides were all within normal limits or negative.

**Table 2:** Laboratory findings on subsequent presentation LDH: lactate dehydrogenase; AST: aspartate aminotransferase; ALT: alanine aminotransferase; PCR: polymerase chain reaction

Laboratory finding	Value	Reference range
Hemoglobin	7.2 g/dL	13-17 g/dL
Platelets	31 K/mcL	140-450 K/mcL
LDH	623 units/L	86-234 units/L
Haptoglobin	<8 mg/dL	30-200 mg/dL
Reticulocyte count	0.3%	0.3-2.5%
Ferritin	5,344 ng/mL	26-388 ng/mL
D-dimer	8.9 mg/L	<0.57 mg/L
AST	34 units/L	<=37 units/L
ALT	64 units/L	<64 units/L
Direct Coombs	Negative	Negative
Indirect coombs	Negative	Negative
Fecal occult blood	Negative	Negative
Paroxysmal nocturnal hemoglobinuria panel	Normal antigenic expression of CD59 on red blood cells. Normal expression of CD24 and CD66b on granulocytes.	Normal antigenic expression of CD59 on red blood cells. Normal expression of CD24 and CD66b on granulocytes.
ADAMTS13 activity	106%	>=67%
Triglycerides	123 mg/dL	<=149 mg/dL
Parvovirus B19 DNA PCR	Not detected	<=0 IU/mL
Quantitative Epstein-Barr virus viral load	Not detected	<=0 IU/mL
Quantitative cytomegalovirus PCR	Not detected	<=0 IU/mL
Initial interleukin-6 level	37.2 pg/mL	<7 pg/mL
Initial interleukin-2 level	10,394 pg/mL	175.3-858.2 pg/mL
Week 8 interleukin-2 level	2,688 pg/mL	175.3-858.2 pg/mL
Week 10 interleukin-2 level	848.9 pg/mL	175.3-858.2 pg/mL

**Figure 2:**
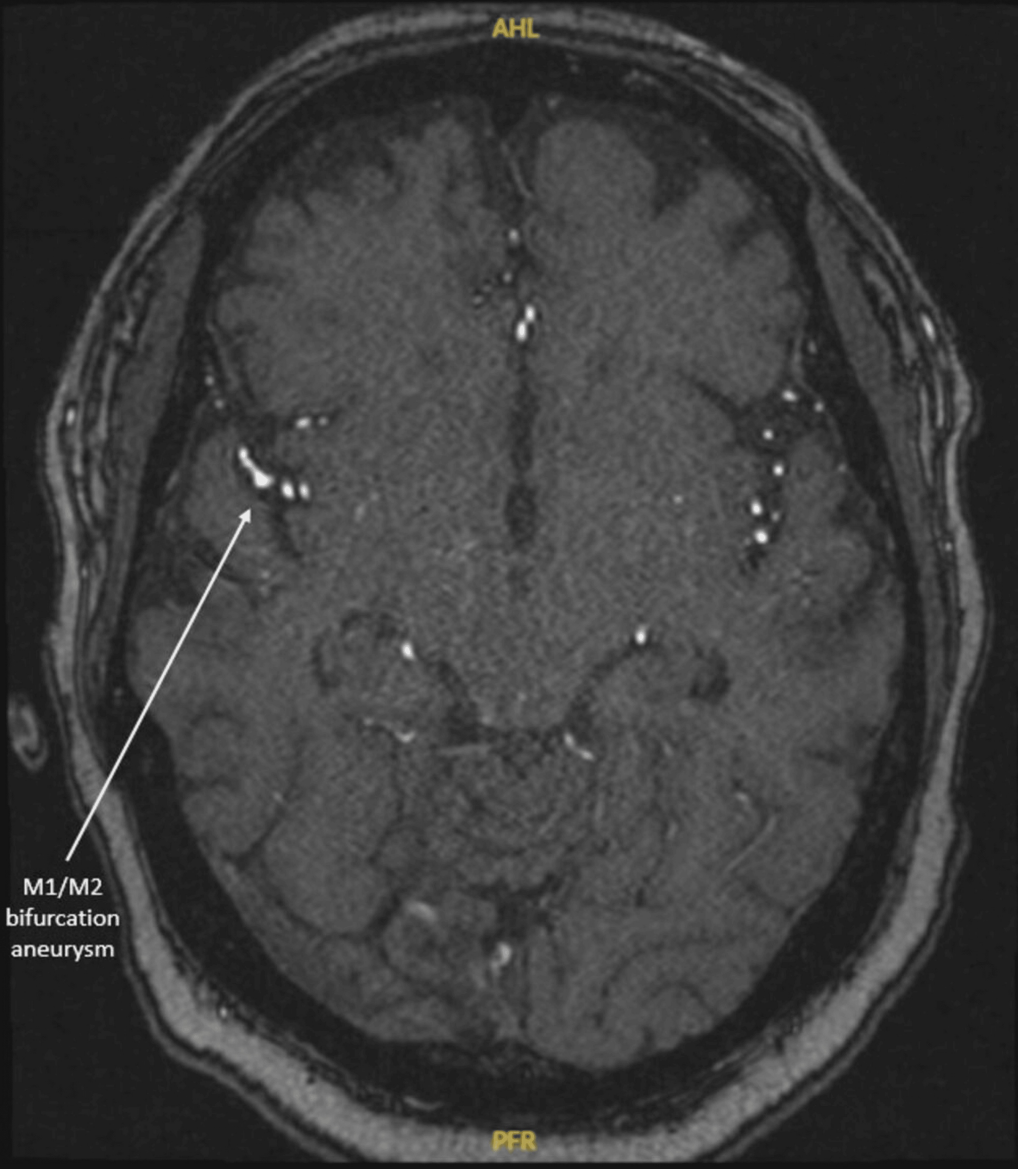
Axial magnetic resonance angiography (MRA) depicting an incidental 2.3 mm M1/M2 bifurcation aneurysm

**Figure 3:**
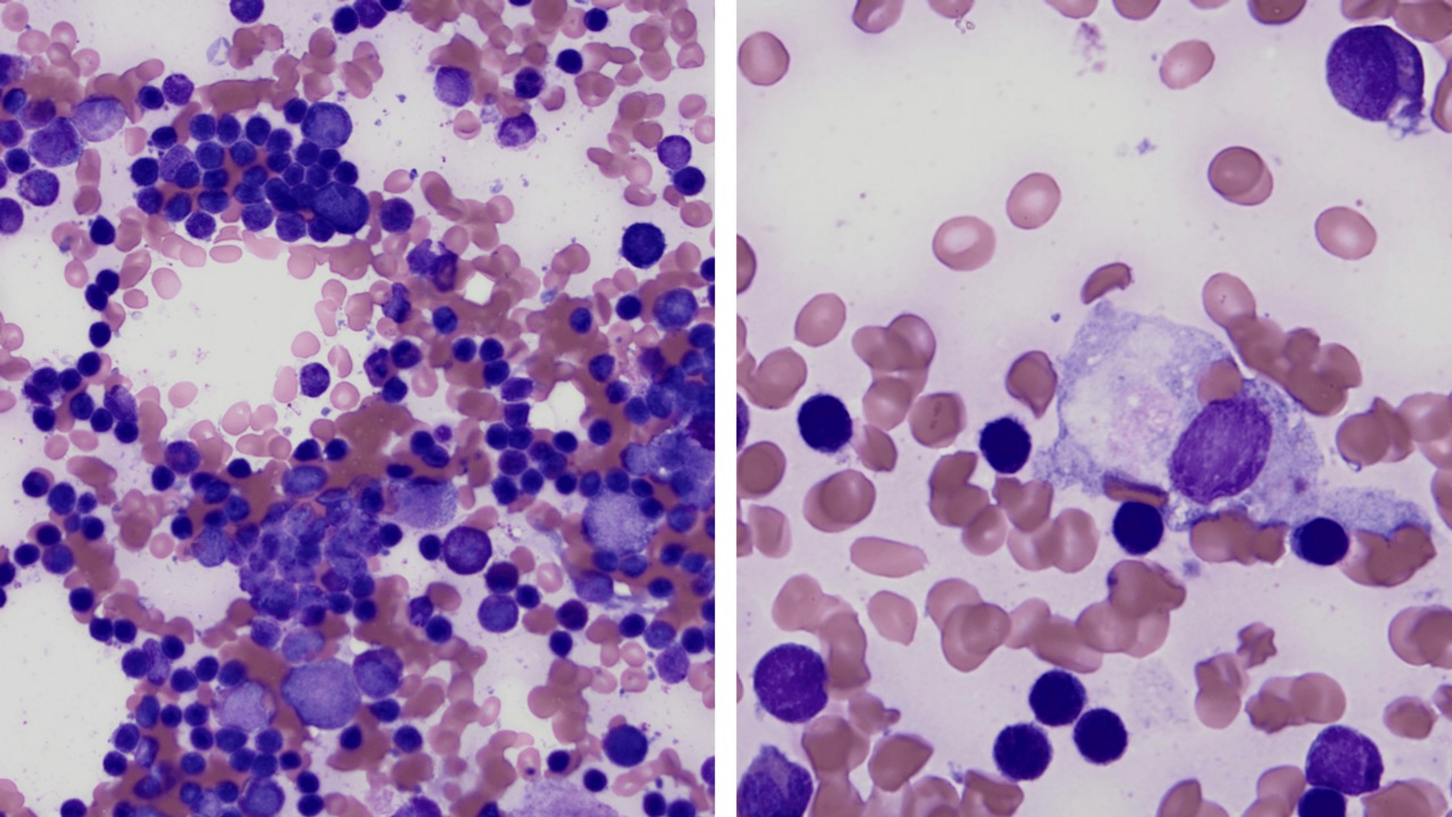
Bone marrow aspirate showing 60% CLL involvement, decreased erythropoiesis, adequate megakaryocytes, and an absence of blasts (left). Single histiocyte exhibiting hemophagocytosis of RBC (right) CLL: chronic lymphocytic leukemia; RBC: red blood cell

Brain magnetic resonance imaging (MRI) and magnetic resonance angiography (MRA) were negative for acute findings but did reveal an incidental 2.3 mm M1/M2 bifurcation aneurysm (Figure 2). CT imaging of the chest, abdomen, and pelvis showed stable splenomegaly with no evidence of CLL progression, so anti-CLL therapy was deferred. Bone marrow biopsy showed 60% CLL involvement, decreased erythropoiesis, adequate megakaryocytes, absence of blasts, and a single histiocyte exhibiting hemophagocytosis of RBCs (Figure 3).

The presence of hemophagocytosis raised suspicion for HLH. Viral studies for Parvovirus B19, Epstein-Barr virus (EBV), and cytomegalovirus (CMV) were unremarkable, ruling out infection-induced HLH. Inflammatory cytokine testing revealed markedly elevated interleukin-2 (IL-2) at 10,394 pg/mL and interleukin-6 (IL-6) at 37.2 pg/mL. Given the timing and findings, nivolumab-induced HLH was suspected. 

Management and Outcomes 

The patient was started on pulse-dose IV dexamethasone (40 mg every six hours for four days), followed by a taper to prednisone 1 mg/kg daily. He developed low-grade fevers and confusion, prompting administration of tocilizumab for suspected CRS and ICANS. He subsequently received anakinra and ruxolitinib. During this time, he was also diagnosed with an *Escherichia* urinary tract infection (UTI) and treated with meropenem. 

**Figure 4:**
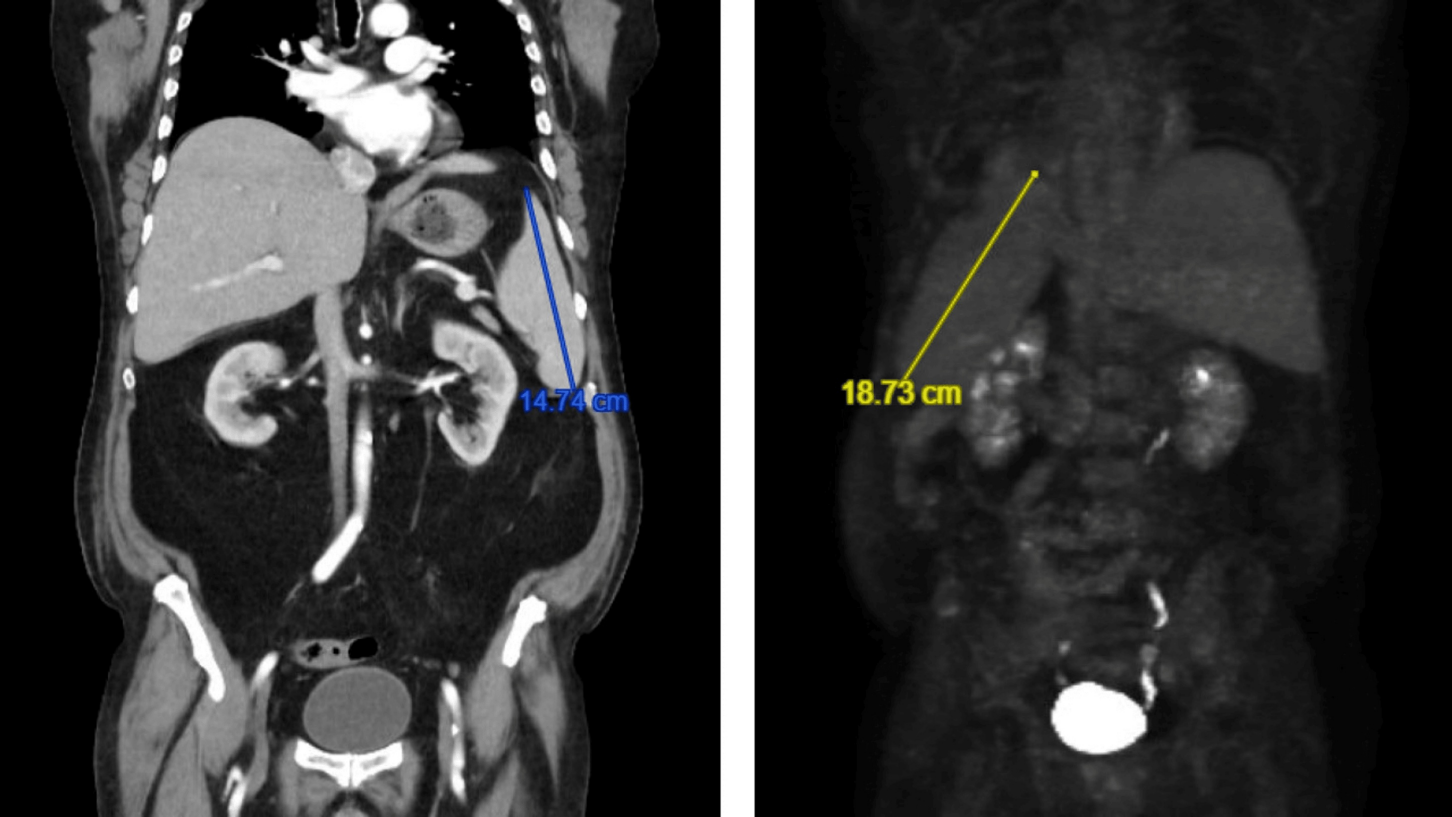
Coronal computed tomography (CT) of the chest, abdomen, and pelvis depicting stable, 14.74 cm splenomegaly (left). Maximal intensity projection from positron emission tomography-computed tomography (PET-CT) depicting worsening, 18.73 cm splenomegaly (right)

Due to persistent symptoms, particularly severe episodic headaches, and suboptimal response, treatment was escalated to the HLH-2004 protocol with etoposide and dexamethasone. Follow-up imaging, demonstrated in Figure 4, revealed worsening splenomegaly, prompting the addition of cyclosporine. Headaches improved after week two of protocol, and inflammatory markers began trending downward. Given clinical improvement and unremarkable MRI brain and CT imaging of the spine, intrathecal methotrexate at week three was deferred. 

The hospital course was complicated by chemotherapy-induced neutropenia, anemia, and thrombocytopenia, which were managed with filgrastim and blood product support. He experienced recurrent infections (including UTIs, prostatitis, and diverticulitis), necessitating prolonged antibiotic therapy. 

After completing eight weeks of induction therapy, he was discharged. At discharge, IL-2 levels had decreased to 2,688 pg/mL. By week 10 of etoposide treatment, IL-2 had normalized to 848.9 pg/mL. 

## Discussion

Literature review

A literature search was performed using PubMed Central to identify reports of HLH associated with PD-1 or PD-L1 inhibitor therapy. The review included case reports, case series, and pharmacovigilance studies published between 2018 and 2024. Search terms included "HLH,” “hemophagocytic lymphohistiocytosis,” “PD-1 inhibitors,” “PD-L1 inhibitors,” and “immune checkpoint inhibitors.” 

Inclusion criteria for the review required that studies specify the PD-1/PD-L1 inhibitor utilized, describe the underlying malignancy, and report the patient’s age, sex, time to HLH onset, method of HLH diagnosis, treatment administered, and clinical outcomes. 

Study Characteristics 

A total of 16 publications were analyzed, including case reports of pembrolizumab-induced HLH, cases of HLH from nivolumab and atezolizumab, pharmacovigilance studies examining HLH risk with ICIs, and retrospective analyses and case series. 

Data extracted from the reviewed literature focused on the type of PD-1/PD-L1 inhibitors administered, underlying malignancy, use of adjuvant therapy in the metastatic setting, patient demographics including age and sex, time to HLH onset, HLH identification, treatment approaches, clinical outcomes and mortality, and author recommendations. 

Patient Demographics and Key Takeaways 

Patient ages ranged from 26 to 86 years, with representation across both sexes. As shown in Table 3, a diverse set of malignancies was reported, including melanoma, lung cancer, breast cancer, glioblastoma, and various hematologic neoplasms. The onset of HLH typically occurred within one to six weeks following initiation of ICI therapy. Most cases involved monotherapy with pembrolizumab or nivolumab, though some reports included combination regimens (e.g., ipilimumab plus nivolumab). Diagnosis was most frequently made using HLH-2004 criteria and/or H-score. 

**Table 3:** List of case reports of hemophagocytic lymphohistiocytosis (HLH) in oncological patients who received immune checkpoint inhibitors HLH: hemophagocytic lymphohistiocytosis; MRI: magnetic resonance imaging; EEG: electroencephalogram

Reference	Authors	Tumor type	Immunotherapy utilized	Onset to HLH	Treatment	Outcomes
[3]	Marar et al.	Squamous cell carcinoma of cutaneous origin	Pembrolizumab	Following 6 cycles of Pembrolizumab	Steroids, tocilizumab, etoposide	Recovery
[4]	Rubio-Perez et al.	Stage IV lung adenocarcinoma	Atezolizumab	2 weeks after initiating Atezolizumab	Steroids, tocilizumab, anakinra, mycophenolate mofetil, etoposide	No clinical improvement. MRI/EEG consistent with diffuse, severe brain involvement
[5]	Zhai et al.	Squamous cell cervical carcinoma	Pembrolizumab	7 days after 1st cycle	Steroids	Recovery
[6]	Kalmuk et al.	Metastatic head and neck squamous cell carcinoma	Pembrolizumab	4 days after cycle 14	Steroids, etoposide	Recovery
[7]	Wei et al.	Thymic carcinoma and non-small cell lung cancer	Pembrolizumab	7 days after Pembrolizumab initiation	Steroids, etoposide	Recovery
[8]	He et al.	Metastatic right atrial angiosarcoma	Toripalimab	After the last round of pazopanib (unclear time period)	Steroids, infliximab, therapeutic plasma exchange	Recovery
[9]	Shah et al.	Metastatic bladder cancer	Pembrolizumab	After 9 months of Pembrolizumab	Steroids, etoposide	Not reported
[10]	Sadaat et al.	Metastatic melanoma	Pembrolizumab	31 days after receiving dose 6 of Pembrolizumab	Steroids	Recovery
[11]	Herman et al.	Sarcomatoid carcinoma of pulmonary origin	Nivolumab	6 months after starting treatment, and 6 days after the most recent nivolumab dose	Steroids, tocilizumab	Recovery
[12]	Thummalapalli et al.	Glioblastoma	Nivolumab	Developed on cycle 2, day 17 of nivolumab	Steroids	Rapid clinical decline
[13]	Noseda et al.	Non-small cell lung cancer, melanoma, renal cell carcinoma, urothelial cancers, and head and neck cancers	Nivolumab, pembrolizumab, atezolizumab, durvalumab, avelumab, ipilimumab	Median onset: 6.7 weeks after starting immunotherapy	Steroids	31 cases were evaluated. 61% recovered
[14]	Patton et al.	Triple negative breast cancer	Pembrolizumab	Developed acute symptoms days after administration of Pembrolizumab	Steroids, tocilizumab	Recovery
[15]	Diaz et al.	Various; specifics not provided. The most common include non-small cell lung cancer, renal cell carcinoma, and melanoma	Pembrolizumab (also includes nivolumab with combination regimens as well)	Average onset 102.2 days after starting immune-checkpoint inhibitor treatment	Most received corticosteroids; select cases also treated with intravenous immunoglobulin, etoposide, and/or tocilizumab	190 total cases. 58.4% favorable outcomes and 15.3% fatal outcomes
[16]	Holmes et al.	Metastatic melanoma	Combination: Ipilimumab + Nivolumab; rechallenged with Nivolumab monotherapy	Two weeks after receiving the first dose of immunotherapy	Steroids	Recovery
[17]	Hantel et al.	Metastatic melanoma	Ipilimumab and Nivolumab	Three weeks after combination therapy initiation	Steroids	Recovery
[18]	Xu et al.	Non-small cell lung cancer, melanoma, bladder cancer, kidney cancer, leukemia, cervical cancer, squamous cell carcinoma, thymic cancer	Various immune-checkpoint inhibitors: anti-PD-1, PD-L1 and CTLA-4 agents	Median onset: 10.3 weeks after ICI therapy	Steroids, cytokine blockers, supportive care	22 of 27 cases with complete recovery

All cases used corticosteroids as first-line treatment. In moderate to severe cases, escalation of therapy included agents such as intravenous immunoglobulin (IVIG), etoposide, anakinra, and/or tocilizumab. Roughly 70-75% of patients achieved recovery. Delays in diagnosis or progression to severe multiorgan failure contributed to significant mortality. 

Across studies, key recommendations emphasized the need for a high index of suspicion for HLH. Early recognition, prompt initiation of treatment, and creating an organized clinical approach to identifying and managing HLH in patients receiving ICIs were deemed necessary. 

Discussion

ICIs have revolutionized the treatment landscape across a range of malignancies, including melanoma, non-small cell lung cancer, renal cell carcinoma, and Hodgkin lymphoma. By targeting regulatory pathways via blockade of PD-1, PDL-1, and CTLA-4, ICIs not only target malignant cells but also trigger a unique profile of irAEs due to inadvertently attacking healthy tissue. The most life-threatening form of irAEs is HLH, a hyperinflammatory syndrome characterized by uncontrolled activation of cytotoxic T cells and macrophages. 

Although HLH is deemed to be a very rare irAE, it is likely underrecognized due to its nonspecific presentation and diagnostic complexity, contributing to underreporting and delayed treatment [19]. Findings from this review reveal that the majority of reported HLH cases are associated with PD-1 inhibitors. Most specifically, pembrolizumab and nivolumab are identified as key causative agents, likely reflecting their widespread use in clinical practice. Our case report adds to this growing body of evidence. The risk of HLH appears elevated with combination regimens such as nivolumab plus ipilimumab, though HLH has also been well-documented following monotherapy [15]. 

While no definitive risk factors for ICI-associated HLH have been established, potential contributors include high tumor burden, prior exposure to immunotherapy, concurrent infection, and a history of autoimmune disease [6]. Notably, several cases documented HLH onset after only a single ICI dose, highlighting the unpredictable nature of this adverse event and the importance of early vigilance in clinical monitoring. 

## Conclusions

HLH is a rare but life-threatening irAE associated with ICI therapy. This case of a patient with metastatic melanoma illustrates a rapidly progressing, steroid-refractory HLH that developed after initiation of nivolumab. Despite initial treatment with multiple immunosuppressive agents, including dexamethasone, tocilizumab, anakinra, and ruxolitinib, clinical improvement was only observed after implementation of the HLH-2004 protocol with etoposide and the addition of cyclosporine. HLH presents significant diagnostic challenges due to its nonspecific symptoms and clinical overlap with conditions such as sepsis, CRS, and malignancy-related inflammation. The commonly used HLH-2004 criteria and H-score often lack sensitivity in oncology settings, underscoring the need for a revised, cancer-specific diagnostic approach. 

Early recognition and timely intervention are critical to improving patient outcomes. Clinicians should maintain a high index of suspicion for HLH in patients receiving ICIs who present with persistent fevers, cytopenias, or elevated inflammatory markers. As seen in this case, multidisciplinary collaboration is essential in managing the complex clinical course of HLH. Further research is needed to identify at-risk populations, develop more accurate diagnostic tools, and establish standardized treatment guidelines for ICI-associated HLH. As immunotherapy use continues to grow, the development of an oncology-specific HLH scoring system will be increasingly important. 
